# A virulent clone of *Devriesea agamarum* affects endangered Lesser Antillean iguanas (*Iguana delicatissima*)

**DOI:** 10.1038/s41598-017-11874-x

**Published:** 2017-10-02

**Authors:** Tom Hellebuyck, Karl Questel, Frank Pasmans, Leen Van Brantegem, Pascal Philip, An Martel

**Affiliations:** 1 Faculty of Veterinary Medicine, Ghent University, Merelbeke, Belgium; 2 Réserve Naturelle de Marine St. Barth, Gustavia, Saint Barthélemy

## Abstract

Infectious diseases affecting wildlife are drivers of global biodiversity loss. Here we report a bacterial threat to endangered wild reptiles. Since April 2011, a severe skin disease has affected free-ranging, endangered Lesser Antillean iguanas (*Iguana delicatissima*) on the French Caribbean island of Saint Barthélemy and we identified *Devriesea agamarum* as the causative agent. The presence of this bacterium was also demonstrated in healthy lizards (anoles) co-inhabiting the island. All isolates from the iguanas corresponded to a single AFLP genotype that until now has exclusively been associated with infections in lizard species in captivity. The clonal relatedness of the isolates and recent emergence of the disease suggest recent arrival of a virulent *D. agamarum* clone on the island. The presence of healthy but infected lizards suggests the presence of asymptomatic reservoir hosts. This is the first description of a bacterial disease that poses a conservation threat towards free-ranging squamates.

## Introduction

The current global sixth extinction wave also affects reptiles, with an estimated 19% of all reptiles considered threatened^1^. Well known drivers are mostly linked to human activities and include habitat fragmentation, alteration and destruction, over exploitation and the introduction of invasive alien species. Infectious diseases are well known to be crucial in amphibian declines^2^. Although a few viral and mycotic infectious diseases have been documented as possible conservation threats to reptile populations^3–5^, the role of infectious diseases as threats to reptile survival is far less understood. Here we report on the emergence of an infectious disease that affects endangered lizards in their natural environment.

The geographic distribution of the Lesser Antillean iguana (*Iguana delicatissima*), is restricted to 10 main islands in the Lesser Antilles. Based on historical range data, the total population of *I. delicatissima* has most likely experienced declines of at least 70% since European contact^6^. Based on available data, an estimate of total population size for *I. delicatissima* across the region is fewer than 26,000 individuals and the existing population is severely fragmented^6^. Habitat destruction, hunting and introduction of invasive competitive species, especially the green iguana (*I. iguana*), have caused a massive decline of the overall population and have driven certain island populations to extirpation during the last decade^6^. Currently, the Lesser Antillean Iguana is considered endangered according to the International Union for Conservation of Nature (IUCN) Red List of Threatened Species (http://www.iucnredlist.org/details/10800/0).

**Figure 1:**
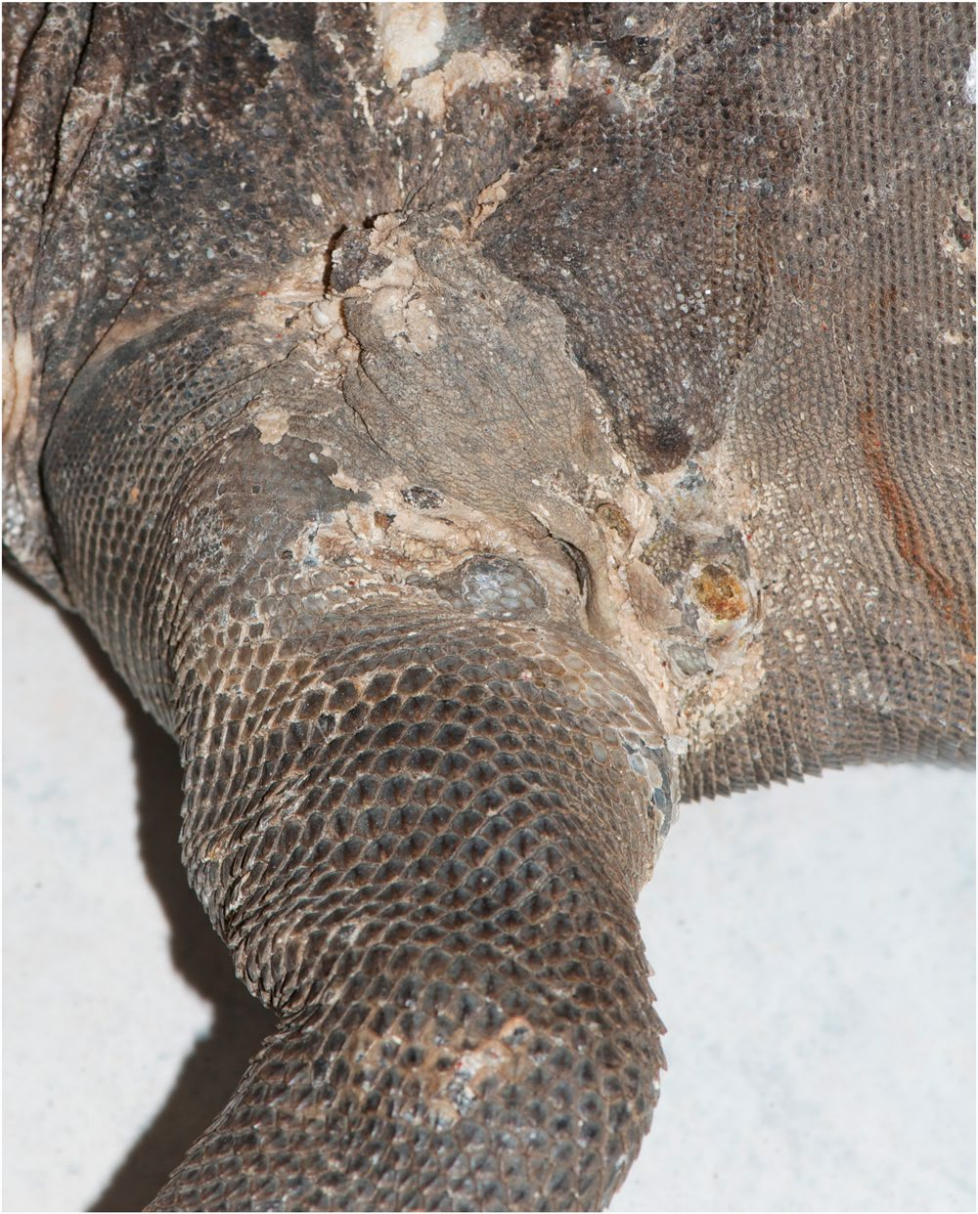
*Devriesea agamarum* associated dermatitis and cutaneous granulomas in the axillar region of a free-ranging Lesser Antillean iguana (*Iguana delicatissima*).

From April 2011 until the end of 2015, 467 free-ranging Lesser Antillean iguanas have been tagged with a passive integrated transponder (PIT) at the French Caribbean island of Saint Barthélemy in order to monitor the population. Approximately 10% of the tagged iguanas showed dermal lesions and affected animals were exclusively males. While most iguanas showed prominent dermatitis with the presence of multiple large cutaneous to subcutaneous skin nodules, some iguanas were seen with less severe multifocal skin masses or superficial dermatitis. Since 2012 until present, five emaciated and two deceased *I. delicatissima* were found showing numerous diffuse dermal nodules covering large areas of the body surface. In all cases, large nodules located at the axillary (Fig. 1) and inguinal region undoubtedly interfered with the ability to show normal foraging and feeding behavior and were considered to have caused a terminal disease state or death in the affected iguanas.

Skin disease in lizards can be caused by a variety of pathogens. In captive lizard populations dermatologic disease is routinely observed and is primarily a consequence of substandard husbandry and underlying disease, facilitating the onset of secondary skin infections^7^. A few bacterial and mycotic agents on the other hand are considered as primary pathogens towards the development of skin disease and may eventually eradicate entire captive lizard collections^8,9^. Over the past decade, several emerging fungal diseases have led to large-scale declines and extinction events in wild ectothermic host populations^2,4,5^. Outbreaks of bacterial infections causing high morbidity in free-ranging populations of reptiles, however, are extremely rare and mostly limited to respiratory infections in chelonians caused by *Mycoplasma* species infection^10,11^. Herein we report the association of *Devriesea agamarum* infection with skin disease in a free-ranging population of *Iguana delicatissima* on Saint Barthélemy.

## Results

### Microbiological examination

Abundant growth of smooth, whitish, small colonies surrounded by a narrow zone of hemolysis was observed following culturing of skin biopsies and skin lesion swabs from all affected *I. delicatissima*. Identical isolates were obtained from the oral cavity of one affected *I. delicatissima*, the cloaca of one healthy *I. delicatissima* and the cloaca of 2 *C. gingivinus*. These isolates were submitted to API^®^ Coryne (bioMérieux, Marcy l’etoile, France) and 16S rRNA gene sequencing as previously described by Martel *et al*.^12^. Based on the results of morphological and biochemical testing the isolates were identified as *Devriesea agamarum*. Following 16S rRNA gene sequencing, 100% similarity with the type strain of *D. agamarum* (IMP2 = LMG 24257^T^) was revealed for all isolates. Mycological cultures did not reveal the presence of clinically relevant fungal isolates.

### Histopathology of skin biopsies

In all sections from the skin biopsies epidermal hyperplasia with orthokeratotic hyperkeratosis, spongiosis as well as adjacent epidermal edema and/or granuloma formation was present. Gram stained sections revealed colonization with Gram-positive bacteria in the superficial corneal layers and throughout the caseous debris in the core of the granulomas. Mild hyperemia and infiltration with heterophils was noted in the dermis of some sections.

### AFLP profiles

The AFLP patterns obtained from two independent experiments showed high similarity. All *D. agamarum* isolates obtained from the healthy and affected *I. delicatissima* and the cloaca of one *C. gingivinus* corresponded to AFLP type C that was previously demonstrated in captive *Uromastyx* species showing cheilitis and/or dermatitis (Table 1, isolate 17). The *D. agamarum* isolate cultured from the cloaca of 2 other *C. gingivinus* corresponded to AFLP type B that has previously been isolated from skin and organ lesions in variety of captive lizards (Table 1, isolate 16) but also proved to be part of the oral microbiota in asymptomatically infected *P. vitticeps*.

**Table 1:** AFLP types of *Devriesea agamarum* isolates obtained from free-ranging Lesser Antillean iguanas (*Iguana delicatissima*) and Anguilla anoles (*Ctenonotus gingivinus*) and captive lizard species (isolate 13–23).

Isolate	Origin	Species	AFLP type
1	abscess	*Iguana delicatissima*	C
2	abscess	*I. delicatissima*	C
3	abscess	*I. delicatissima*	C
4	abscess	*I. delicatissima*	C
6	abscess	*I. delicatissima*	C
7	abscess	*I. delicatissima*	C
8	oral cavity	*I. delicatissima*	C
9	cloaca	*I. delicatissima*	C
10	cloaca	*Ctenonotus gingivinus*	C
11	cloaca	*C. gingivinus*	B
12	cloaca	*C. gingivinus*	B
13	oral cavity	*Pogona vitticeps*	A
14	oral cavity	*P. vitticeps*	A
15	oral cavity	*Eublepharis macularius*	A
16	dermatitis/liver	Agama impalearis*	B
17	dermatitis	*Uromastyx dispar*	C
21	dermatitis	*Crotaphytus collaris*	F
22	dermatitis	*P. vitticeps*	G
23	dermatitis	*P. vitticeps*	H

The isolates from captive lizards were collected between 2003 and 2009 to study the relatedness of *D. agamarum* isolates using AFLP as described by Devloo *et al*.^13^.

*D. agamarum type strain IMP2 (LMG 24257).

## Discussion

The gross pathological and histopathologic features of the skin lesions combined with the isolation of *D. agamarum* in all affected *I. delicatissima* deliver strong evidence for a primary etiological role of the bacterium in the development of skin disease in the free-ranging iguanas. In one affected iguana and 3 healthy lizards, *D. agamarum* was isolated from the oral cavity and cloaca respectively. The latter corresponds to previous reports documenting *D. agamarum* to occur as a part of the oral and cloacal microbiota in healthy lizards^13–15^.

The occurrence of 8 different AFLP types demonstrate pronounced variation of *D. agamarum* strains in captive lizards^13^. Interestingly, all isolates from the iguanas corresponded to a single AFLP genotype that until now has exclusively been associated with dermatitis and/or cheilitis in captive *Uromastyx* species^13^. These findings may indicate clonal expansion of a virulent *D. agamarum* strain in free-ranging *I. delicatissima*. In addition, the presence of AFLP type B and C in asymptomatically infected, free-ranging lizards demonstrates that the latter may act as a persistent source of clinical infection in at least *I. delicatissima*.

Worldwide *D. agamarum* causes large scale disease in captive lizard collections and is primarily characterized by severe dermatitis or granulomas sometimes resulting in septicaemia and death^12,15,16^. Especially desert dwelling lizard species seem to be susceptible to *D. agamarum* associated disease, severely compromising their successful captive maintenance^13,15^. Persistency of the disease within lizard collections is largely promoted by the presence of asymptomatic carriers and long-term environmental survival leading to high morbidity and mortality, depending on the lizard species involved^8,13,14^. Recently, the presence of *D. agamarum* has been documented in healthy as well as clinically infected non-desert dwelling lizards belonging to the Family Agamidae and the Superfamily Iguanidae^14,17^. Clinical signs described in the latter reports mainly comprise subcutaneous granulomas, identical to those observed in free-ranging *I. delicatissima*. In chronically infected captive lizards, the presence of large dermal granulomas eventually interferes with normal foraging and feeding behavior and may ultimately lead to high mortality rates if left untreated^12,15,16^. This corresponds to the outcome of chronic *D. agamarum* associated disease that was observed in emaciated and deceased *I. delicatissima*. To the best of the authors’ knowledge this is the first description of a bacterial disease posing a conservation threat in free-ranging population of a squamate species.

Based on the distribution of the observed skin lesions in the iguanas, bite-prone regions seem most frequently affected. As *D. agamarum* is considered a part of the oral microbiota in healthy lizards, wound infection following biting lesions, e.g. resulting from territorial combat, may result in the development of bacterial granulomas as observed in free-ranging *I. delicatissima*. The latter may explain why all iguanas with skin lesions sampled in this study were exclusively males. Indeed, a breach of skin integrity has been documented to play an important role towards the onset of clinical infection with *D. agamarum* and other dermal pathogens in reptiles^15,18,19^. It remains unclear, however, why *D. agamarum* associated skin disease has not been previously reported to cause high morbidity, taking into account that several endemic lizard species were readily identified as asymptomatic carriers in this study. Until the importance of altered environmental factors, impaired wild host defense mechanisms or decisive virulence factors of the involved *D. agamarum* isolates are revealed, the history of the disease in free-ranging iguanas will remain unclear.

The rapid spread of non-native green iguanas seems to be the most recent and urgent threat to the Lesser Antillean iguana population^6^. Large numbers have been intentionally or accidentally released as a consequence of the international pet trade^20^. Besides competition for food and territory, green iguanas readily interbreed with the Lesser Antillean iguana raising fundamental problems towards conservation management as genetic purity of certain animals becomes uncertain^6^. Moreover, *D. agamarum* has been demonstrated to be part of the oral microbiota in a variety of lizard species in captivity^13^, including healthy green iguanas (personal communication, Hellebuyck T.). For this reason, invasive species, especially those originating from the international pet trade, may form a vessel for the introduction of *D. agamarum* in free ranging populations. Except for the single hybrid iguana, we unfortunately were unable to sample green iguanas during the present study.

Although an effective antimicrobial treatment is available to cure *D. agamarum* dermatitis in captive lizards and autovaccination was shown to protect against *D. agamarum* associated septicemia^21^, establishing effective therapeutic or preventive measures in affected free-ranging iguanas would be highly challenging. Moreover, the presence of asymptomatically infected lizards and the long-term environmental survival of the bacterium may severely contribute to persistency in affected populations. As the remaining island populations are severely fragmented, individuals have been translocated to augment local populations. Taking into account the high morbidity of the disease in free-ranging iguanas, the screening for the presence of *D. agamarum* associated skin lesions in iguanas should be implemented as an additional but highly important control strategy towards establishing a conservation action plan that aims to ensure long-term survival of the species throughout its natural range.

The association of *D. agamarum* with the skin lesions observed in this population of *I. delicatissima* indicates a new possible threat for this endangered lizard species and has not previously been reported in iguanids of the Western Hemisphere. As this is the first time that *D. agamarum* associated skin lesions have been observed in the St. Bartélemy population, this might be considered as a newly introduced disease.

## Materials and Methods

### Sample collection

A total of 32 lizards were captured on the island of Saint Barthélemy and examined for the presence of skin lesions. Sampling of the lizards was authorized and approved by the local enforcement authority (Collectivité d’outre-mer de Saint Barthélemy) and performed in accordance with relevant guidelines and regulations. The obtained samples were transported in accordance with CITES regulations (CITES certificate No. FR1597100054-K). The examined lizards comprised 10 Anguilla bank ameivas (*Pholidoscelis plei*), 5 Anguilla anoles (*Ctenonotus gingivinus*), 16 *I. delicatissima* and 1 *I. delicatissima* x *I. iguana* hybrid. All *I. delicatissima* were fitted with a subcutaneous PIT to allow the collection of individual life history and movement data for individual iguanas, the *I. delicatissima* x *I. iguana* hybrid was euthanized after the sampling. While all 8 affected iguanas and the single, healthy hybrid were males, the 8 remaining healthy iguanas were females.

In 7 *I. delicatissima* multiple skin lesions were present with a variable distribution. The skin of the neck, lateral body wall, legs and base of the tail were the most frequently affected skin sites (Fig. 1). The skin lesions mainly consisted of large, firm subcutaneous to cutaneous nodular lesions and/or dermatitis. While an intact epidermis covered most nodular lesions, some skin lesions showed a yellowish epidermis with a hyperkeratotic, encrusted aspect. The skin nodules consisted of a thick capsule and caseous core.

Oral and cloaca swabs were collected from all examined lizards. In addition, swabs and full thickness skin biopsies were collected from skin lesions in the affected *I. delicatissima* for microbiological and histopathological examination. Tissues for histopathology were collected in formalin, embedded in paraffin followed by hematoxylin and eosin staining after sectioning.

Oral and cloacal swabs as well as swabs collected from skin lesions and skin biopsies were cultured on Columbia agar with 5% sheep blood (COL, Oxoid GmbH, Wesel, Germany) and colistin nalidixic acid agar with 5% sheep blood (CNA agar, Oxoid GmbH) during 24–48 h at 37 °C and 5% CO_2_. For isolation of fungi, swabs from skin lesions and skin biopsies were placed on Sabouraud dextrose agar (SAB agar, Oxoid GmbH) and observed for fungal growth over a 14-day period at 25 °C.

### Analysis of Amplified Fragment Length Polymorphism (AFLP) profiles and comparison to previously described *D. agamarum* AFLP genotypes from captive lizards

AFLP was performed for all obtained *D. agamarum* isolates and compared to 8 *D. agamarum* isolates from captive lizards representing 6 AFLP genotypes as previously demonstrated by Devloo *et al*.^13^ (Table 1).

**Figure 2:**
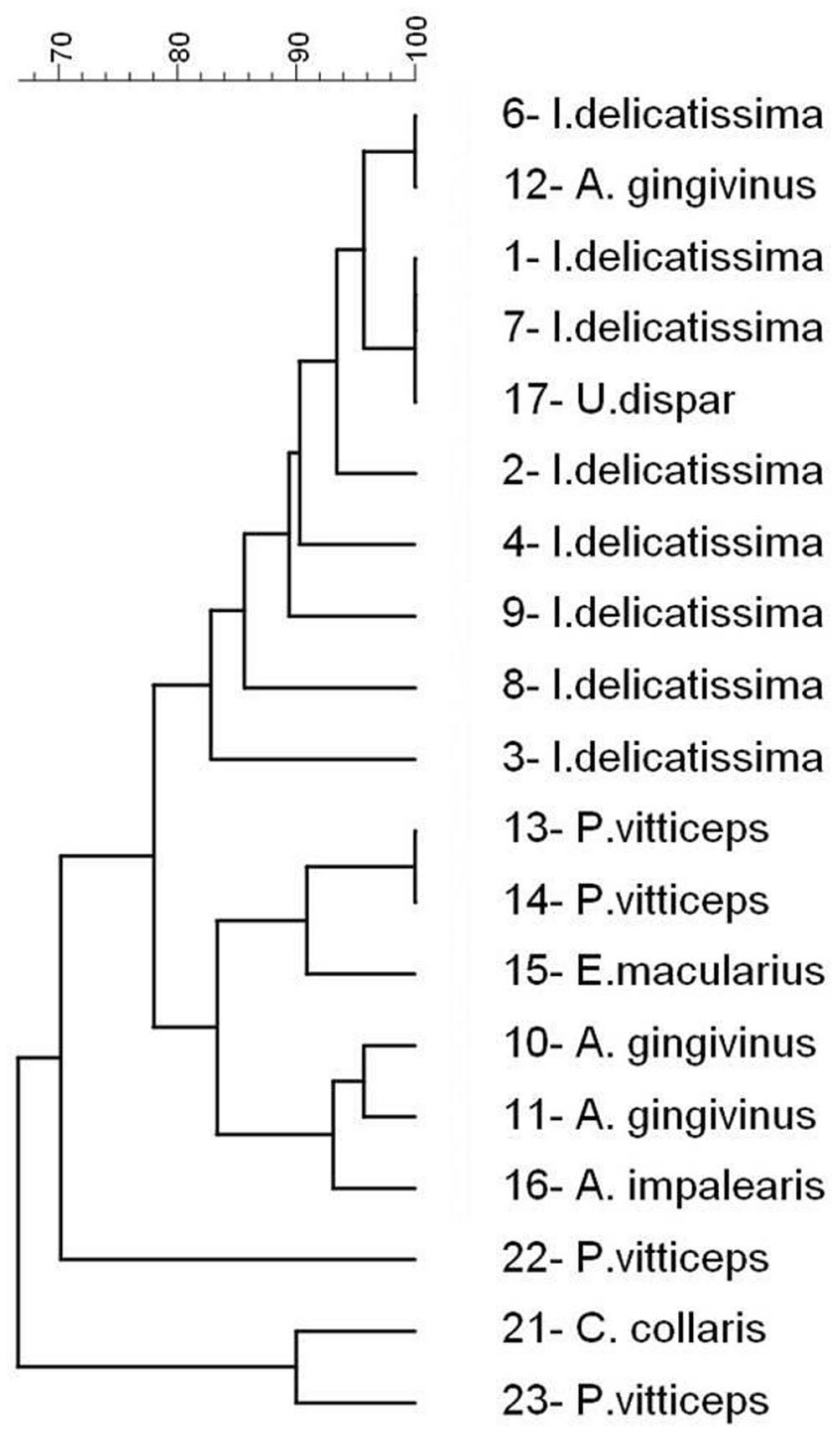
Dendrogram constructed based on the obtained AFLP profiles of *Devriesea agamarum* isolates. Cluster analysis was performed with UPGMA using DICE algorithm and a tolerance and optimization level of 1.06%. The strains were classified in the same AFLP type if the relatedness was higher than 70%.

The pictures of the gels were imported as TIFF-files into Bionumerics version 4.6 (Applied Maths, Sint-Martens-Latem, Belgium) and a similarity matrix was calculated according to the DICE algorithm using optimization settings and tolerance level of 1.06. A UPGMA (Unweighted Pair Group Method with Arithmetic Mean) dendrogram (Fig. 2) was constructed based on the average similarity matrices of two replicates. Isolates were classified in the same AFLP type if their relatedness was higher than 70%. A three dimensional visualisation was created using the multidimensional scaling tool based on the average similarity matrix.

### Data availability

All data generated or analysed during this study are included in this published article.
